# Unraveling breast cancer response to neoadjuvant chemotherapy through integrated genomic, transcriptomic, and circulating tumor DNA analysis

**DOI:** 10.1186/s13058-025-02026-5

**Published:** 2025-05-01

**Authors:** Menghao Dong, Jian Chen, Nannan Lu, Song Wang, Wenhui Wei, Ziming Wang, Jinnan Wang, Jinguo Zhang, Xinghua Han, Fufeng Wang, Qiuxiang Ou, Hua Bao, Xiaopeng Ma, Benjie Shan, Yueyin Pan

**Affiliations:** 1 Department of Clinical Oncology, Division of Life Sciences and Medicine, The First Affiliated Hospital of USTC, University of Science and Technology of China, No. 17 Lujiang Road, Luyang District, Hefei, Anhui 230001 China; 2 Nanjing Geneseeq Technology Inc., Nanjing, Jiangsu 210032 China; 3 Department of Breast Surgery, Division of Life Sciences and Medicine, The First Affiliated Hospital of USTC, University of Science and Technology of China, No. 17 Lujiang Road, Luyang District, Hefei, Anhui 230001 China

**Keywords:** Pathological complete response, Neoadjuvant chemotherapy, Breast cancer, Molecular biomarkers

## Abstract

**Introduction:**

Neoadjuvant chemotherapy (NAC) is a standard treatment for breast cancer (BC) to shrink tumors and facilitate surgery. However, the molecular underpinnings of response to NAC and prognosis have not been well characterized.

**Methods:**

We enrolled 73 stage II/III BC patients who received NAC followed by surgery. Tumor tissue samples were available from 36 patients at baseline and 38 at the time of surgery. Plasma circulating tumor DNA (ctDNA) was collected at three time points: before NAC (*n* = 63), during NAC (*n* = 42), and after NAC (*n* = 40). Comprehensive genomic, transcriptomic, and ctDNA analyses were performed to identify biomarkers associated with pathological complete response (pCR) and survival outcomes.

**Results:**

Nine baseline mutations, including *DNHD1* and *PLEC*, along with HIPPO pathway alterations, were associated with pCR. Responsive tumors exhibited immune activation and downregulated PI3K-Akt and AGE-RAGE pathways, while non-pCR tumors showed reduced cytokine and immune receptor activity. Undetectable ctDNA during and after NAC was predictive of treatment efficacy and correlated with improved survival. Baseline mutations in *USH2A* were associated with shorter disease-free survival (hazard ratio: 11.9; 95% confidence interval: 2.8–50.8; *P* < 0.001), with a consistent trend observed for overall survival. Elevated *NHSL1* expression in baseline tumors indicated an initial treatment response but was later associated with tumor relapse and poor overall survival (*P* = 0.026 and *P* = 0.023, respectively), findings that were validated in an independent clinical cohort (*N* = 30) through immunohistochemistry staining.

**Conclusion:**

Our comprehensive multi-omics analysis identified promising biomarkers predictive of treatment response and survival in BC patients receiving NAC followed by surgery. These findings underscore the importance of early tumor assessment for improved patient stratification and prognostication.

**Supplementary Information:**

The online version contains supplementary material available at 10.1186/s13058-025-02026-5.

## Introduction

Breast cancer (BC) is the second-leading cause of cancer-related mortality in females worldwide [1]. Neoadjuvant chemotherapy (NAC) is widely accepted as the standard treatment for locally advanced BC and a standard option for primary operable disease to downgrade tumors and make radical surgery more feasible [2]. From a clinical perspective, NAC provides a unique opportunity to evaluate tumor response, allowing for a more rapid assessment of the efficacy of new therapeutic agents. In cases of resistance to systemic treatments, promptly adjusting the dose or switching to another agent during NAC, as well as employing escalation or de-escalation strategies in the adjuvant setting based on the response to NAC, can minimize toxicity and side effects, ultimately improving patient survival outcomes through individually tailored treatments.

The complete remission of all viable residual tumor cells, confirmed by histopathologic examination of the removed breast and axillary tissue, also known as pathological complete response (pCR), is considered evidence of the complete eradication of the locoregional disease after NAC and a potential surrogate marker of long-term survival [3, 4]. Previously established predictors of response to NAC include small tumor size, high tumor grade, high proliferation rate, tumor necrosis, and the presence of tumor-associated lymphocytes [5]. In addition, patients present with triple-negative breast cancer (TNBC) and those with hormone receptor-negative (HR-) and HER2-positive (HER2 +) tumors often exhibit a better response to NAC [6, 7]. However, this advantage does not always translate into long-term survival, as HER2 + and TNBC patients who fail to respond to therapy have a worse outcome compared to patients with HR + tumors [8]. Since some patients who respond to currently available chemotherapeutic agents may also experience disease recurrence at a later stage, more efforts should be made to better characterize the molecular makeup of patients to identify reliable biomarkers that predict NAC response and survival outcomes.

In this study, we aim to identify biomarkers associated with tumor response and long-term survival in stage II/III BC patients treated with NAC followed by curative-intent surgery. Whole-exome sequencing (WES) was utilized to characterize the mutational landscape of patients at baseline, while whole transcriptome sequencing (WTS) was used to explore the relationships between gene expression, NAC response, and survival outcomes. Plasma samples were collected at three preoperative time points, including P1 (before NAC), P2 (on-NAC), and P3 (post-NAC), to assess the feasibility of undetectable ctDNA as a predictor of pCR.

## Materials and methods

### Patients and samples

In this retrospective study, we included a total of 73 patients with resectable stage II/III BC who were admitted to the participating hospital between 2017 and 2022 for neoadjuvant chemotherapy followed by curative-intent surgery. The primary inclusion criteria were as follows: i) age between 18 and 70 years; ii) histologically confirmed stage II/III BC deemed resectable after investigator assessment; iii) measurable tumor lesions with a sum of diameters ≥ 10 mm on magnetic resonance imaging (MRI) scans; iv) an Eastern Cooperative Oncology Group performance status score of 0 to 1; v) no prior systemic treatments, such as radiotherapy and endocrine therapy; and vi) for patients with occult BC, pathologically confirmed measurable lesions in axillary lymph nodes. Key exclusion criteria included: i) histologically confirmed metastatic BC; ii) presence of uncontrolled cardiovascular or cerebrovascular diseases, coagulation disorders, connective tissue diseases, or severe infectious diseases; iii) prior systemic anti-cancer therapy; iv) lactating or pregnant patients; v) psychiatric disorders or poor compliance; vi) other malignancies, except for cured basal cell carcinoma of the skin or cervix carcinoma in situ; vii) left ventricular ejection fraction < 50%; and viii) unavailability of pathological assessment records and survival data.

Patients in this study received one of the following NAC regimens: EC-T (epirubicin and cyclophosphamide followed by paclitaxel), EC-TH (epirubicin and cyclophosphamide followed by paclitaxel and trastuzumab), TEC (paclitaxel, epirubicin, and cyclophosphamide), AC-TH (doxorubicin and cyclophosphamide followed by paclitaxel and trastuzumab), or EC-TCb (epirubicin and cyclophosphamide followed by paclitaxel and capecitabine). Adjuvant treatment strategies included: i) hormone therapy for 5 years; ii) targeted therapy for 1 year; iii) a combination of targeted therapy for 17 cycles (approximately a year) followed by continued hormone therapy for 5 years. Local radiation therapy was administered according to CSCO guidelines to eliminate residual cancer cells and reduce local recurrence risk.

Treatment-naïve tumor samples were obtained by core-needle biopsy, and post-NAC tumors were collected at surgery, both subjected to WES and WTS analysis. Plasma samples were collected preoperatively at three landmark time points for ctDNA profiling by targeted next-generation sequencing (NGS): before NAC/baseline (P1), on-NAC (P2, by the completion of the first cycle of chemotherapy), and post-NAC (P3). Clinical staging of primary tumors was done according to the American Joint Committee on Cancer guidelines (AJCC, 8th edition). Radiological imaging of tumor response was defined using RECIST 1.1 criteria [9], comparing imaging data of local/regional tumors obtained during NAC and before surgery with baseline images. To verify the prognostic impact of genetic variants, we retrieved clinical and corresponding mutation data for the BRCA-Metabric (*N* = 223) [10] from cBioPortal (https://www.cbioportal.org/). Inclusion criteria required the availability of both disease-free survival (DFS) and overall survival (OS) data, as well as relevant clinical characteristics, including female sex, receipt of chemotherapy, and mastectomy as the surgical procedure. Clinical samples, with linked pseudo-anonymized clinical information of patients, were obtained with appropriate ethical approval from the relevant institutional review board (Ethics Approval Number: 2019KY086). All study procedures were conducted in accordance with the principles of the Helsinki Declaration. All patients provided written informed consent to participate and for publication.

### Pathological assessment

Pathological response was assessed for the percentage of residual viable tumors in the breast and axillary lymph nodes, irrespective of ductal carcinoma in situ (ypTO/is ypNO) that were identified based on conventional hematoxylin and eosin staining of surgical specimens under light microscopy [5]. Residue disease following NAC was quantified using the residue cancer burden (RCB) index, which was categorized into RCB classes: RCB-0 (no residual invasive disease), RCB-I (RCB score 0–1.36), RCB-II (1.37–3.28), and RCB-III (> 3.28), corresponding to pCR, minimal, moderate, and extensive residual disease, respectively [11].

### Sample processing and DNA library construction

Formalin-fixed paraffin-embedded (FFPE) samples underwent de-paraffinization with xylene, and genomic DNA extraction was conducted using the QIAamp DNA FFPE Tissue Kit (Qiagen Cat. No. 56404) following the manufacturer’s instructions. The plasma fraction of the peripheral blood was isolated within two hours after specimen collection, which was first centrifuged at 1,800 g for 10 min, followed by circulating cell-free DNA extraction and purification using the QIAamp Circulating Nucleic Acid Kit (Qiagen Cat. No. 55114). Extraction of genomic DNA from white blood cells in sediments for each specimen was performed using the DNeasy Blood and Tissue Kit (Qiagen Cat. No. 69504), subsequently utilized as the normal control. Genomic DNA was quantified using the dsDNA HS assay kit on a Qubit 3.0 fluorometer (Life Technology, US), followed by NGS library construction using the KAPA Hyper Prep kit (KAPA Biosystems). Exome enrichment capture was performed using the xGen Exome Research Panel v2 and Hybridization and Wash Reagents Kit (Integrated DNA Technologies). Hybridization-based target enrichment was carried out using xGen lockdown probes targeting 437 pan-cancer genes (GeneseeqPrime®, Nanjing Geneseeq Technology Inc., Nanjing, China). Library fragment size was analyzed on a Bioanalyzer 2100 using the High Sensitivity DNA Kit (Agilent Technologies, Santa Clara, CA). Sequencing was performed on the enriched libraries on the Illumina HiSeq4000 platform.

### Somatic genetic variant mutation calling in tumor samples

Quality control was performed on FASTQ files using Trimmomatic [12]. Reads with a quality reading below 20 and/or N bases were removed. Paired-end reads were then aligned to the reference human genome hg19 using the Burrows-Wheeler Aligner (BWA, https://github.com/lh3/bwa/tree/master/bwakit). Picard (https://broadinstitute.github.io/picard/) was used to remove PCR duplications, and local alignment around indels and base quality score recalibration was performed using GATK3 (https://software.broadinstitute.org/gatk/). Tumor purity was estimated using the ABSOLUTE algorithm [13] (Additional file 1). All baseline tumor biopsies included for variant calling had an estimated tumor purity of at least 0.20, ensuring adequate tumor cellularity for reliable somatic mutation detection. Somatic single nucleotide variant (SNV) and insertion/deletions (indels) calling were performed using Mutect2 [14]. Variants were first filtered based on the following criteria: i) absence in matched genomic DNA from white blood cells; ii) absence in > 1% of the population in the 1000 Genomes Project or the Exome Aggregation Consortium 65,000 exomes database. To be considered a true variant, candidate SNVs and indels needed to meet the following criteria: i) variant allele frequency (VAF) ≥ 0.5% and supporting reads ≥ 3 for hotspot mutations (≥ 20 mentions in COSMIC v92 [15]); VAF ≥ 1% and supporting reads ≥ 6 for non-hotspot mutations. The final compilation of mutations went through a manual review process using the Integrative Genomics Viewer. Copy number variation (CNV) analysis was performed using FACETS [16]. A CNV event on a given chromosome was classified as “chromosomal” if at least 60% of its segments exhibited consistent copy number alteration. Focal CNVs were characterized as amplifications or deletions based on the total copy number and ploidy calculated by the FACETS algorithm [17]. The curated variant list was further refined to focus on somatic alterations within ten canonical signaling pathways representing common cancer hallmarks [18].

### Plasma ctDNA profiling

A plasma sample was defined as ctDNA-positive if at least one somatic genetic variant was detected using the pan-cancer NGS panel (GeneseeqPrime™, Nanjing Geneseeq Technology Inc., Nanjing, China). For a variant to be considered eligible, it had to meet the following criteria: i) absent in matched genomic DNA from white blood cells after excluding clonal hematopoiesis; ii) not present in > 1% of the population in the 1000 Genomes Project or the Exome Aggregation Consortium 65,000 exomes database; iii) VAF ≥ 0.1% with at least 3 supporting reads for SNVs and indels; iv) a gene ratio cutoff of ≥ 1.6 for copy number gain and < 0.6 for copy number loss; and v) split-reads ≥ 3 for structural variants.

### Preparation of RNA-seq libraries

Total RNA was isolated from FFPE samples using the miRNeasy FFPE Kit (Qiagen, Valencia, CA). When available, RNA and DNA were extracted in parallel from adjacent tissue sections of the same FFPE tumor blocks to ensure consistency across genomic and transcriptomic analyses. The quantity and quality of the total RNA were assessed using NanoDrop 2000 (Thermo Fisher Scientific, Waltham, MA) and an Agilent 2100 Bioanalyzer (Agilent Technologies, Santa Clara, CA), respectively. Libraries were prepared from extracted RNA using the KAPA Stranded mRNA-Seq Kit (KAPA Biosystems, Wilmington, MA), per the manufacturer’s instructions using 1 µg of RNA with at least an RNA integrity number of 7. The pooled libraries were paired-end sequenced on the Illumina NovaSeq6000 platform.

### RNA sequencing data analysis

RNA-seq data in FASTQ files underwent initial demultiplexing and quality control using Trimmomatic [12]. BWA was then used to remove transfer RNA and ribosomal RNA reads. Adaptor and primer sequences, as well as short reads less than 20 bp were also excluded. Mapping to the human reference genome hg19 was performed using STAR (version 2.7.3a) with default parameters [19], which was followed by isoform and gene level quantification using RSEM (version 1.3.0) [20]. Protein-coding genes with a Transcripts Per Million (TPM) value exceeding 0.5 in more than 25% of samples were included in downstream analysis. Differentially expressed genes (DEGs) were analyzed using the R package “DESeq2”. Unless otherwise specified, the thresholds for identifying DEGs were log2 (fold change) >  ± 1, and a false discovery rate (FDR)-adjusted *P*-value (padj) < 0.05. Pathway enrichment analysis of Gene Ontology (GO), Kyoto Encyclopedia of Genes and Genomes (KEGG), and Gene Set Enrichment Analysis (GSEA) was carried out using the R package “ClusterProfiler”. High expression was characterized as the top quartile of patients exhibiting the highest expression and low expression as the other three quartiles. PAM50 classification was performed using the “molecular.subtyping” function from the R package “genefu” [21].

The immune cell infiltration levels in the tumor microenvironment (TME) were determined by the relative proportion of twenty-two cell populations by the CIBERSORT (cell-type identification by estimating relative subsets of RNA transcripts) method [22], and MCP-counter (Microenvironment Cell Populations-counter) algorithm using default parameters [23]. TME scoring was estimated using principal component analysis based on gene expressions associated with different TME phenotypes using the R package “TMEscore” (https://github.com/DongqiangZeng0808/TMEscore) [24–26]. In brief, the TMEscoreA is immune cell-related, characterized by genes implicated in immune activation and response to viruses and IFNγ. TMEscoreB is stromal cell-related and enriched for genes involved in extracellular matrix remodeling, epithelial-mesenchymal transition, cell adhesion, and angiogenesis [24]. The sum of these two relevant individual scores was termed TMEscore.

### Immunohistochemistry (IHC) analysis

Validation of NHSL1 protein expression was performed on baseline tumor samples from an independent clinical validation cohort of 30 BC patients who underwent NAC at our hospital between January 2015 and December 2022. FFPE samples were deparaffinized using xylene and rehydrated through a graded series of alcohol solutions. Endogenous peroxidase activity was inhibited by treating the sections with 3% hydrogen peroxide for 15 min. The tissue sections were then blocked with 10% goat serum at room temperature for 40 min and subsequently incubated overnight at 4 °C with an anti-NHSL1 antibody (#PAB22375, Abnova) in a 1:500 dilution. Following this, the sections were incubated with a biotin-labeled anti-rabbit secondary antibody at room temperature for 1 h. After thorough washing, the sections were stained with 3, 3-diaminobenzidine, and counterstained with hematoxylin.

NHSL1 expression was evaluated using an integrated scoring method based on staining intensity and the percentage of positive cells [27]. Staining intensity was graded as follows: negative (0 points), light yellow/weak positive (1 point), yellowish-brown/positive (2 points), and dark brown/strong positive (3 points). The percentage of positive cells was categorized as follows: ≤ 25% (1 point), 26%-50% (2 points), 51%-75% (3 points), and > 75% (4 points). The final IHC score was calculated by multiplying the staining intensity and percentage scores. Patients with an IHC score ≤ 7 were defined as having low NHSL1 expression, while those with a score > 7 were categorized as having high NHSL1 expression.

### Statistical analysis

All statistical analyses were performed in R (version 4.1.3). Fisher’s exact test was used to compare the frequencies of categorical variables between groups, while the Wilcoxon rank-sum test was used to compare the distribution of continuous data. Cohen’s Kappa coefficients were calculated to assess the concordance between subtype classifications based on the conventional histological method and sequencing-based profiling. The Point-Biserial test was used to assess the correlation between gene expression (continuous variable) and pathological response (dichotomous variable). Receiver operating characteristic (ROC) curves were generated for genes correlated with pCR, using thresholds of correlation coefficient >  ± 0.5 and *P*-value < 0.05. The performance of undetectable ctDNA in predicting pCR was characterized by sensitivity, specificity, positive predictive value (PPV), and negative predictive value (NPV), based on the counts of true positives, true negatives, false positives, and false negatives. Univariate logistic regression models were used to assess the association between clinical variables and pCR, with odds ratios (ORs) and 95% confidence intervals (CIs) reported. For genomic alterations, we focused on variants with a mutation frequency ≥ 15% for biomarker exploration of both baseline and surgical tumor samples. Protein-coding genes that passed the initial filter with a TPM exceeding 0.5 in more than 25% of samples were included in downstream survival analyses. Disease-free survival (DFS) was calculated as the duration from surgery to tumor relapses or patient death, while overall survival (OS) was determined based on the time from initial diagnosis to patient death. For patients lost to follow-up, OS was calculated based on the time of the last follow-up. Kaplan–Meier survival curves were used to compare the survival of subgroup patients, with statistical differences assessed using the log-rank test. Cox proportional hazard models were fitted to estimate hazard ratios (HRs) with 95% CIs, and the proportionality of hazards was assessed using log(-log) survival plots. A two-sided *P*-value of less than 0.05 was considered significant for all tests unless otherwise indicated (*P < 0.05, **P < 0.01, ***P < 0.001).

## Results

### Patient characteristics

**Fig. 1:**
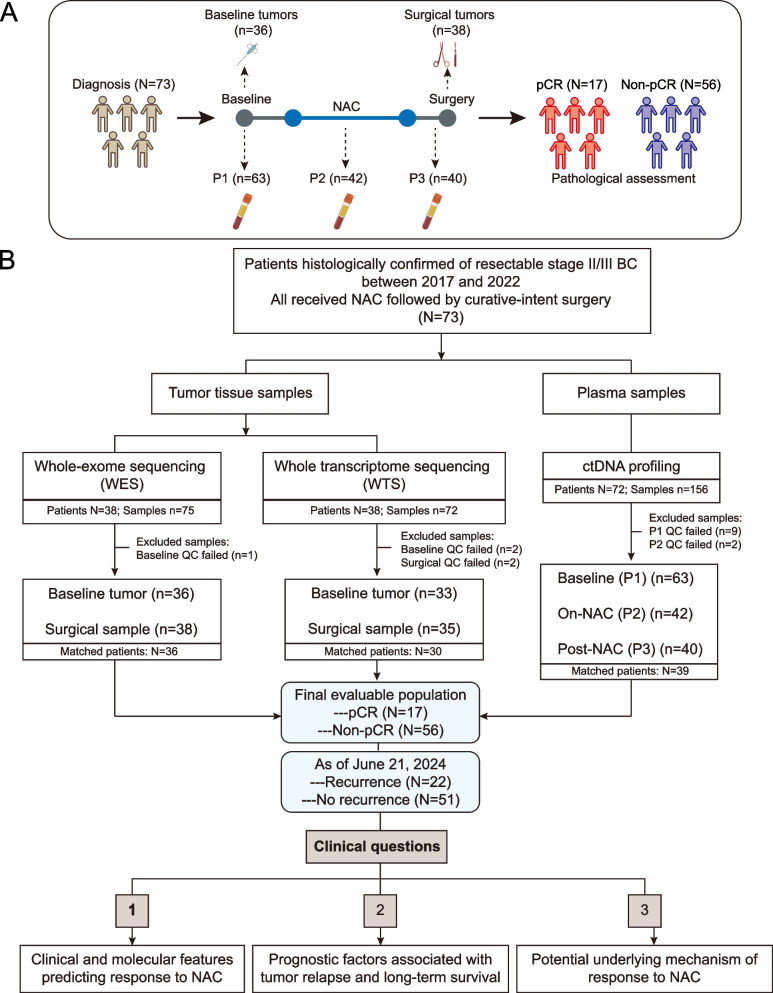
Inclusion of patients in subanalyses. **A** Overview of sample collection for downstream analyses. **B** Flow diagram of patient inclusion, with clinical questions addressed by each analysis indicated. BC, breast cancer; NAC, neoadjuvant chemotherapy; pCR, pathological complete response; QC, quality control; NA, not available

Seventy-three patients with stage II/III breast cancer were included in this retrospective study, all of whom received neoadjuvant chemotherapy (NAC) followed by curative-intent surgery (lumpectomy or modified radical mastectomy) (Fig. 1). Tumor samples were obtained either through biopsy or during surgery, and surgical specimens were assessed for pathological response. Preoperative plasma samples were collected at three time points: before NAC (P1), during NAC (P2), and after NAC (P3), to evaluate the association between ctDNA status and tumor response to NAC. Sample availability for each patient is shown in Supplementary Material: Figure S1.

The median age was 49 years (interquartile range [IQR]: 45–58 years), with about half above 50 years old (Table 1). Two-thirds of patients presented with clinical stage II, and 80.8% (59/73) had nodal involvement at the initial diagnosis. Approximately half of the patients received local regional radiotherapy post-surgery, and 75.3% (55/73) received systemic adjuvant therapies, including 34 with hormone therapy, 6 with targeted therapy, and 15 with a combination of hormone and targeted therapies. Pathological assessment of viable residual tumor cells in resected breast tumors and axillary lymph nodes classified patients into pCR (*N* = 17) and non-pCR (*N* = 56). Tumor response to NAC was associated with clinical stage (*P* = 0.007) and lymph node metastasis (*P* = 0.002) (Table 1). Univariate logistic regression model analysis indicated that patients with TNBC (odds ratio [OR] = 4.77, 95% confidence interval [CI]: 1.21–21.41, *P* = 0.03) and HR + /HER2 + subtypes (OR = 3.75, 95% CI: 0.84–17.96, *P* = 0.08) were more likely to achieve pCR compared to those with the HR + /HER2- subtype (Supplementary Material: Figure S2A). As of June 21, 2024, the median follow-up was 58.2 months (IQR: 55.2–85.9 months), with 22 patients (30.1%, 22/73) experiencing disease recurrence and 10 patients (13.7%, 10/73) deceased (Supplementary Material: Figure S2B). Patients who achieved pCR demonstrated longer disease-free survival (DFS) and overall survival (OS) (Supplementary Material: Figure S2C, D).

**Table 1:** Clinical characteristics of patients

	All (*N* = 73)	pCR (*N* = 17)	Non-pCR (*N* = 56)	*P*-value
Age				0.269
Median (IQR)	49 (45–58)	46 (39–51)	51 (45–60)	
< 50 years	37 (50.7%)	11 (64.7%)	26 (46.4%)	
≥ 50 years	36 (49.3%)	6 (35.3%)	30 (53.6%)	
Subtype				0.098
HR + /HER2 +	15 (20.5%)	5 (29.4%)	10 (17.9%)	
HR + /HER2-	34 (46.6%)	4 (23.5%)	30 (53.6%)	
HR- /HER2 +	6 (8.2%)	1 (5.9%)	5 (8.9%)	
TNBC	18 (24.7%)	7 (41.2%)	11 (19.6%)	
Clinical stage				0.007**
II	49 (67.1%)	16 (94.1%)	33 (58.9%)	
III	24 (32.9%)	1 (5.9%)	23 (41.1%)	
Primary tumor (T)				0.935
T1	9 (12.3%)	2 (11.8%)	7 (12.5%)	
T2	47 (64.4%)	12 (70.6%)	35 (62.5%)	
T3	16 (21.9%)	3 (17.6%)	13 (23.2%)	
T4	1 (1.4%)	0 (0.0%)	1 (1.8%)	
Regional lymph nodes (N)				0.004**
N0	14 (19.2%)	8 (47.1%)	6 (10.7%)	
N1	43 (58.9%)	9 (52.9%)	34 (60.7%)	
N2	11 (15.1%)	0 (0.0%)	11 (19.6%)	
N3	5 (6.8%)	0 (0.0%)	5 (8.9%)	
Distant metastases (M)				~
M0	73 (100.0%)	17 (100.0%)	56 (100.0%)	
NAC regimen				0.287
EC-T	49 (67.1%)	10 (58.8%)	39 (69.6%)	
EC-TH	13 (17.8%)	3 (17.6%)	10 (17.9%)	
TEC	5 (6.8%)	3 (17.6%)	2 (3.6%)	
AC-TH	3 (4.1%)	1 (5.9%)	2 (3.6%)	
EC-TCb	3 (4.1%)	0 (0.0%)	3 (5.4%)	
ADT regimen				0.141
No systemic ADT	18 (24.7%)	7 (41.2%)	11 (19.6%)	
Hormone therapy	34 (46.6%)	4 (23.5%)	30 (53.6%)	
Targeted therapy	6 (8.2%)	1 (5.9%)	5 (8.9%)	
Hormone + targeted	15 (20.5%)	5 (29.4%)	10 (17.9%)	
Radiotherapy				0.012*
Yes	38 (52.1%)	4 (23.5%)	34 (60.7%)	
No	35 (47.9%)	13 (76.5%)	22 (39.3%)	

*Abbreviations*: *IQR* Interquartile range, *NAC* Neoadjuvant chemotherapy, *ADT* Adjuvant therapy, *pCR* Pathological complete response, *EC-T* Epirubicin and cyclophosphamide followed by paclitaxel, *EC-TH* Epirubicin and cyclophosphamide followed by paclitaxel and trastuzumab, *TEC* Paclitaxel, epirubicin, and cyclophosphamide, *AC-TH* Doxorubicin and cyclophosphamide followed by paclitaxel and trastuzumab, *EC-TCb* Epirubicin and cyclophosphamide followed by paclitaxel and carboplatin

### Mutational landscape of patients and its association with tumor response and prognosis

**Fig. 2:**
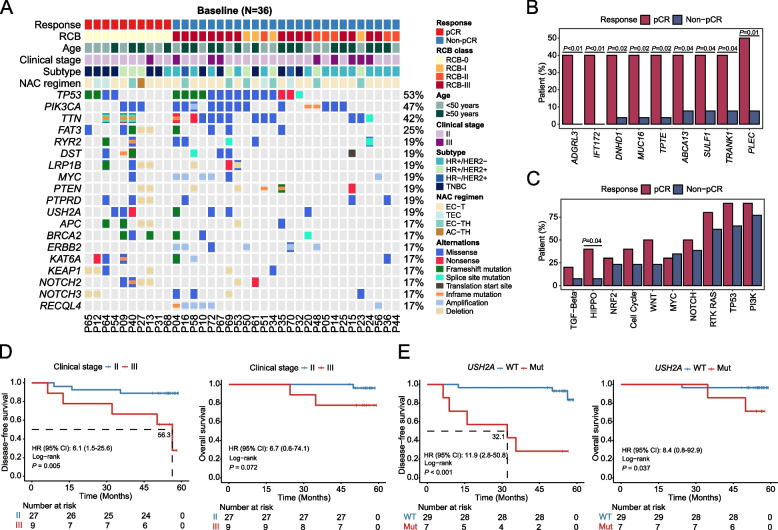
Mutational landscape of patients before and after NAC. **A** Co-mutation plot of oncogenic genetic variants in baseline tumors. **B**, **C** Bar plots showing the proportion of patients harboring specific mutations (**B**) and genetic variations categorized into ten oncogenic signaling pathways in baseline tumors (**C**). **D**, **E** Kaplan–Meier curves demonstrate the disease-free survival and overall survival of patients stratified by clinical stage (**D**) and baseline *USH2A* mutations (**E**)

Whole-exome sequencing (WES) was performed on baseline tumor samples from 36 patients (Fig. 1B). *ERBB2* copy number variation identified by WES showed 100% concordance with IHC-defined HER2 status, supporting the reliability of genomic sequencing and its alignment with standard clinical assessment (Supplementary Material: Figure S3A). At baseline, the most frequently mutated genes, regardless of pathological response, were *TP53* (53%), *PIK3CA* (47%), and *TTN* (42%) (Fig. 2A; Supplementary Material: Figure S4A). *TTN*, due to its exceptionally large coding region, is prone to a high mutation frequency across various cancer types [28, 29]. While *TTN* mutation count has been proposed as a surrogate marker for tumor mutational burden (TMB) in hypermutated tumors such as melanoma and colorectal cancer, its correlation with TMB is markedly weaker in cancers with lower mutation rates, including breast, kidney, and thyroid cancers [28]. In this context, *TTN* mutations in BC are more likely to represent passenger events; however, further functional analyses are needed to confirm their biological significance. Patients with pCR had a significantly higher prevalence of mutations in *ADGRL3*, *IFT172*, *DNHD1*, *MUC16*, *TPTE*, *ABCA13*, *SULF1*, *TRANK1*, and *PLEC*, and genetic variants related to the HIPPO signaling pathway (Fig. 2B, C). Copy number variant analysis showed no significant differences in arm- or focal-level copy number changes between pCR and non-pCR patients, consistent with the lack of difference in chromosome instability score (Supplementary Material: Figure S4B-D).

Given the challenge of tumor recurrence after surgery, we explored biomarkers for identifying high-risk patients. Univariate Cox hazard models revealed significant associations between clinicopathological features and DFS, including clinical stage and five gene mutations with ≥ 15% mutational frequency (Supplementary Material: Table S1). After multivariate correction, clinical stage and *USH2A* mutations remained significant, indicating an independent association with DFS despite the relatively small sample size (Fig. 2D, E; Supplementary Material: Figure S4E). Similar trends were observed within the BRCA-Metabric dataset, though statistical significance was not reached, possibly due to differences in ethnicity, BC subtypes, and treatments (Supplementary Material: Figure S4F). To understand the potential underlying mechanisms highlighting the prognostic impact of baseline *USH2A* mutations, we analyzed the expression profiles of these patients. Using thresholds of log2 (fold change) >  ± 1 and padj < 0.1, we identified 8 upregulated and 2 downregulated genes between baseline tumors with or without *USH2A* mutations (Supplementary Material: Figure S4G). Among these, *MSL1* was the only differentially expressed gene meeting the stringent cutoff of padj < 0.05 in the gene expression analysis.

### Elevated NHSL1 expression in pCR patients is associated with worse survival outcomes

**Fig. 3:**
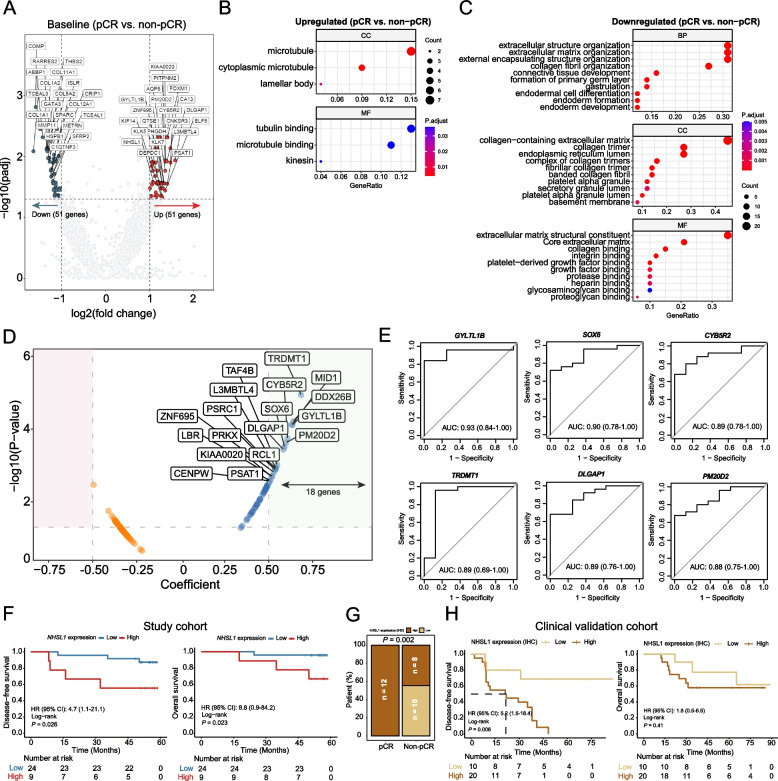
Gene expression profiling of baseline tumors. **A** Volcano plot showing differentially expressed genes (DEGs) in pathological complete response (pCR) tumors compared to non-pCR tumors. **B**, **C** Functional annotation of upregulated (**B**) and downregulated genes (**C**) in pCR tumors via GO analysis. **D** Correlation of gene expression with pCR defined by TPM values. **E** ROC curves assessing the prediction of pCR using TPM expression levels of the top six significantly associated genes. **F** Kaplan–Meier curves demonstrate the disease-free survival and overall survival of subgroup patients categorized by *NHSL1* expression levels in baseline tumors. **G** The proportion of pCR and non-pCR patients with high or low NHSL1 protein expression in the clinical validation cohort, assessed by immunohistochemistry. **H** Kaplan–Meier curves showing the disease-free survival and overall survival of the clinical validation cohort stratified by baseline NHSL1 protein expression. GO, Gene Ontology; TPM, Transcripts Per Million; ROC, receiver operating characteristic

Similarly, concordance analysis of subtype classification between conventional IHC and RNA sequencing-based PAM50 profiling showed strong agreement, with a Kappa coefficient of 0.813 (*P* < 0.001) and 87.9% (29/33) of patients classified identically by both methods (Supplementary Material: Figure S3B). Baseline gene expression levels were compared between pCR (*n* = 8) and non-pCR (*n* = 25) tumors, which identified 102 DEGs (Fig. 3A). While the heatmap of all gene expressions did not perfectly classify patients, the DEG’s expression matrix successfully clustered pCR and non-pCR tumors into two distinct groups (Supplementary Material: Figure S5A). Functional analysis revealed that upregulated genes in baseline pCR tumors were likely involved in tubulin binding and pathways related to amino acid metabolism, mismatch repair (MMR), and mTOR signaling (Fig. 3B; Supplementary Material: Figure S5B). Conversely, downregulated genes were significantly associated with extracellular matrix organization, the PI3K-Akt pathway, and the AGE-RAGE signaling pathway (Fig. 3C). Correlation analysis identified 18 genes whose expression was positively associated with pCR (Fig. 3D). The individual ability of baseline DEGs to identify patients with tumors that may achieve pCR at surgery ranges from 0.76–0.93, with *GYLTL1B* (Glycosyltransferase-Like 1B, also known as *LARGE2*) showing the highest AUC of 0.93 (95% CI: 0.84–1.00) (Fig. 3E). The complete AUC list for the remaining genes is provided in Supplementary Material: Table S2. Gene expression profiles were also used to analyze the immune cell composition within the tumor microenvironment (TME). While no significant differences were observed in TME scores between pCR and non-pCR tumors, CIBERSORT analysis revealed that baseline pCR tumors had a significantly higher fraction of memory B cells and resting natural killer (NK) cells, but a lower proportion of activated mast cells in the TME (Supplementary Material: Figure S5C, D). Additionally, non-pCR tumors exhibited higher levels of cancer-associated fibroblasts (CAFs), which may be linked to poorer NAC responses (Supplementary Material: Figure S5E).

Interestingly, univariate Cox hazard models demonstrated that baseline Nance-Horan Syndrome-like 1 (*NHSL1*) expression, although upregulated in pCR tumors, was significantly associated with shorter DFS and OS (Fig. 3F; Supplementary Material: Table S3). In the multivariate analysis, *NHSL1* expression remained a significant independent predictor of shorter DFS and OS, irrespective of pathological response (*P* = 0.046 and *P* = 0.043, respectively) (Supplementary Material: Figure S5F, G). To validate these findings, we analyzed an independent clinical validation cohort (*N* = 30) and quantified NHSL1 protein expression through immunohistochemistry (IHC). Notably, all pCR patients exhibited high NHSL1 expression, compared to 44.4% (8/18) in the non-pCR group (*P* = 0.002) (Fig. 3G). Furthermore, high NHSL1 protein expression was significantly associated with DFS (hazard ratio: 5.2; 95% CI: 1.5–18.4; *P* = 0.006), with a consistent trend observed for OS (Fig. 3H). Together, these intriguing findings suggest that higher NHSL1 expression in baseline tumors may reflect an initial response to NAC; however, it is likely associated with an overall unfavorable prognosis in BC patients treated with NAC followed by surgery.

### Molecular subtyping identifies patients at higher risk of disease recurrence and poor survival

Single biomarkers may not fully capture the intricate interplays within the TME, highlighting the need for identifying combined biomarkers through multi-omics analysis. Here, we classified patients into three molecular subtypes based on the mutational status of *USH2A* and the transcriptional level of *NHSL1* in baseline tumors (Supplementary Material: Figure S6A). Type I included patients with wild-type *USH2A* and low *NHSL1* expression, while Type III consisted of patients with concurrent *USH2A* mutations and high *NHSL1* expression. Patients with either of these two unfavorable prognostic biomarkers were categorized as Type II. As expected, type III patients exhibited the shortest DFS and OS compared to the other two subtypes (*P* < 0.001 and *P* = 0.002, respectively) (Supplementary Material: Figure S6B). Furthermore, molecular subtype III remained a significant prognostic factor for poorer clinical outcomes, independent of pathological response (Supplementary Material: Figure S6C). However, given the limited number of patients, especially those with both baseline *USH2A* mutations and high *NHSL1* expression, future studies with larger cohorts and external datasets are essential to validate these findings.

### Potential underlying mechanisms of NAC in breast cancer

**Fig. 4:**
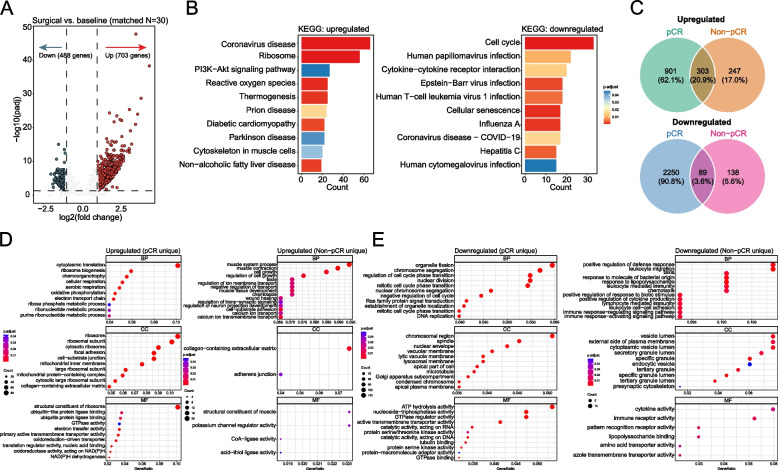
Transcriptional changes before and after NAC. **A** Volcano plot showing differentially expressed genes (DEGs) in surgical tumors versus baseline tumors from 30 matched patients. **B** Functional analysis of upregulated and downregulated genes in surgical samples via KEGG. **C** Venn diagrams display the overlapping and uniquely identified upregulated (top) or downregulated (bottom) genes in pCR and non-pCR patients. **D**, **E** Gene Ontology analysis of uniquely upregulated (**D**) and downregulated (**E**) genes in patients categorized by pathological response

Next, we analyzed gene expression changes before and after NAC using matched tumor samples from 30 patients. There were 703 upregulated and 488 downregulated genes identified through WTS (Fig. 4A). KEGG analysis revealed that the significantly upregulated genes were enriched in the PI3K-Akt signaling and reactive oxygen species pathways, while downregulated genes were associated with the cell cycle pathway and cytokine-cytokine receptor interactions (Fig. 4B). We further profiled DEGs based on pathological response status of patients. As shown in Fig. 4C, 303 upregulated and 89 downregulated genes were commonly identified in pCR (*n* = 6) and non-pCR (*n* = 24) patients. GO analysis of these DEGs yielded results consistent with those from the KEGG analysis of matched patients (Supplementary Material: Figure S7A, B).

We subsequently characterized DEGs that were exclusively identified in either pCR or non-pCR patients to explore potential mechanisms of action of NAC. In pCR patients, upregulated genes after NAC were significantly enriched in cytoplasmic translation, ribosome biogenesis, and chemoorganotrophy (Fig. 4D). In contrast, upregulated genes in non-pCR samples were mainly involved in muscle system process, regulation of cell growth, and chemotaxis. Uniquely downregulated genes in pCR samples were associated with the regulation of cell cycle phase transition and DNA replication, while those in non-pCR samples were related to leukocyte-mediated immunity and immune receptor activity, providing a potential explanation for their limited response to NAC (Fig. 4E).

The immune cell contexture was then compared between surgical and baseline samples. TMEscoreA, represented by the expression of gene signatures associated with immune response, was significantly higher in baseline samples, irrespective of pathological response (Supplementary Material: Figure S7C). Analysis of MCP-counter scores for immune cell populations revealed that changes in cytotoxic cell populations may be associated with NAC responses. In patients achieving pCR, there was a significant reduction in cytotoxic cell populations, including CD8 + T cells, NK cells, neutrophils, and endothelial cells after NAC (Supplementary Material: Figure S7D). In contrast, although non-pCR patients also showed a decrease in these populations, the changes were not statistically significant. Collectively, the patterns of immune cell composition before and after NAC align with DEG profiling results, suggesting that immune response modulation plays a critical role in the antitumor activity of neoadjuvant chemotherapy.

### Undetectable ctDNA during and after NAC reliably predicts treatment efficacy

**Fig. 5:**
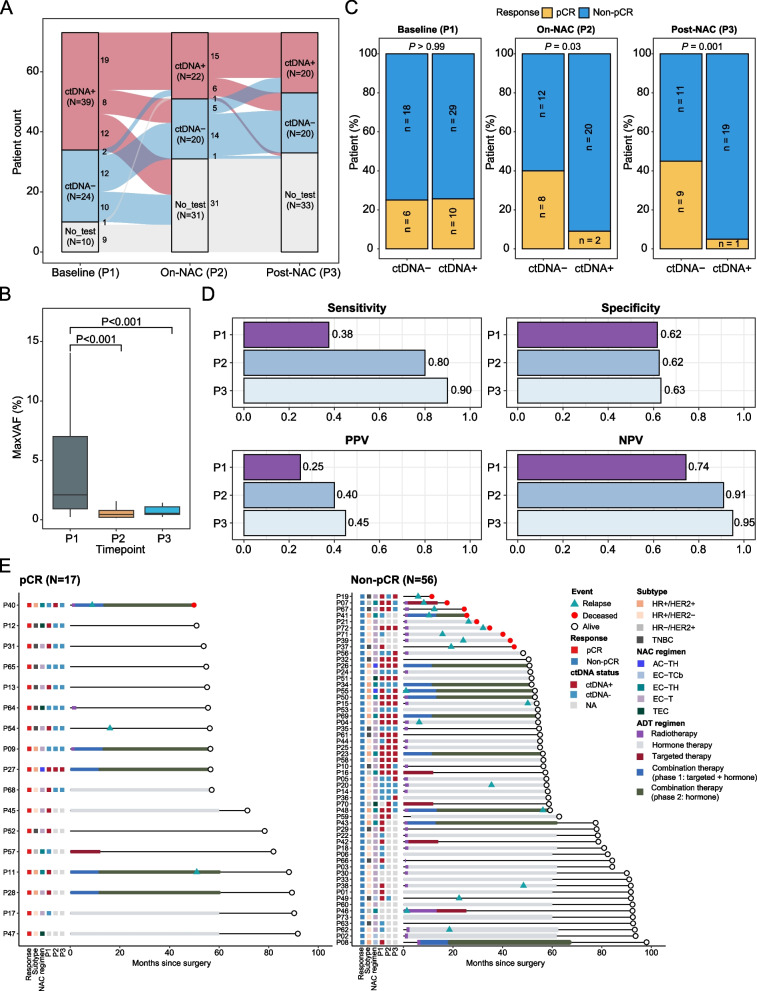
ctDNA dynamics during NAC associated with tumor response. **A** Sankey plot depicting changes in the proportion of patients stratified by ctDNA status at baseline, on-NAC, and post-NAC time points. **B** Box plot showing the change in the maximum variant allele frequency (maxVAF) in plasma ctDNA during NAC. **C** Bar plots show the proportion of patients achieving pathological complete response (pCR) or non-pCR, stratified by ctDNA status at the three time points. **D** Performance analysis evaluating the predictive value of undetectable ctDNA at each of the three time points for assessing tumor response to NAC. **E** Swimming plot depicting the timing of tumor recurrence, patient death, and receipt of perioperative treatment regimens, stratified by pathological response. NAC, neoadjuvant chemotherapy; ADT, adjuvant therapy; NA, not available; PPV, positive predictive value; NPV, negative predictive value

The feasibility of preoperative plasma ctDNA and the appropriate time in predicting pCR were evaluated at three time points: at baseline/before-NAC (P1), on-NAC (P2), and after-NAC (P3). The positive ctDNA detection rate was 61.9% (39/63), 52.4% (22/42), and 50% (20/40) at P1, P2, and P3 time points, respectively (Fig. 5A). For patients with matched samples (*N* = 39), the ctDNA detection rates significantly dropped from 64.1% at P1 to 51.3% at P3 (Supplementary Material: Figure S8A). The maximum variant allele frequency (maxVAF) at P2 showed a notable decline compared to P1, reflecting reduced tumor burden during NAC (Fig. 5B; Supplementary Material: Figure S8B).

Dynamic ctDNA changes during chemoradiotherapy have the potential to predict clinical outcomes for locally advanced non-small cell lung cancer patients [30]. However, whether this holds true for BC patients undergoing neoadjuvant chemotherapy remains underexplored. Our study showed that pCR patients were mostly those with undetectable ctDNA during and after NAC (Fig. 5C; Supplementary Material: Figure S8C). The sensitivity of undetectable ctDNA for predicting pCR after NAC was 0.38 (95% CI: 0.15–0.65) at P1, 0.80 (95% CI: 0.44–0.97) at P2, and 0.90 (95% CI: 0.55–1.00) at P3 (Fig. 5D). The overall specificity was similar across all three time points, with P3 reaching a slightly higher specificity of 0.63 (95% CI: 0.44–0.80). Consistent performance was observed in patients with matched samples (Supplementary Material: Figure S8D). The timing of tumor recurrence, death, receipt of perioperative treatments, and ctDNA positivity status for each patient are shown in Fig. 5E. Non-responders exhibited a trend of higher recurrence risk compared to pCR patients, though the difference was not significant due to the limited sample size. Our findings indicate that both landmark and longitudinal ctDNA positivity during NAC, either from P2 to P3 or P1 to P3 time points, were associated with poorer clinical outcomes (Supplementary Material: Fig. S9). However, the prognostic value of on-treatment ctDNA status requires further investigation in larger cohorts.

## Discussion

In this study, we comprehensively characterized the molecular landscape of resectable stage II/III BC in the context of NAC, integrating genomic, transcriptomic, and ctDNA profiling from patient subsets within a cohort of 73 individuals. To the best of our knowledge, this represents one of the largest cohorts to date aimed at identifying predictive and prognostic biomarkers associated with NAC response and survival in BC, based on multi-dimensional molecular data.

Pathological complete response is a US Food and Drug Administration (FDA)-approved surrogate endpoint for DFS and OS in randomized clinical trials evaluating neoadjuvant therapies for BC. Favorable long-term outcomes are typically observed in pCR patients, particularly in aggressive subtypes such as TNBC and HER2 + [31]. However, the surrogacy value of pCR is debated [31–33]. For instance, a meta-analysis comprising 54 randomized clinical trials by Conforti et al. reported a weak association between pCR and survival outcomes [33]. These conflicting findings may arise from varying definitions of pCR (ypTO ypNO vs. ypTO/is ypNO vs. ypTO/is) and the heterogeneity of BC subtypes. Further research into the molecular characteristics of BC is essential for identifying biomarkers that predict NAC response and long-term survival. In this study, we employed WES and WTS for mutational and transcriptional profiling of BC tumors and analyzed plasma-derived ctDNA at preoperative time points to gain additional clinical insights.

Evaluating tumor response to NAC provides a great opportunity to assess the efficacy of therapeutic agents and optimize treatment strategies, maximizing survival benefits while minimizing toxicity and side effects. Through WES, we identified nine mutations and alterations in the HIPPO pathway that were more frequently detected in baseline tumor samples from pCR patients. Among these, *DNHD1* (Dynein Heavy Chain Domain 1), predicted to be involved in cilium movement, was found to co-occur with mutations in *TTN* and *MUC* genes in BC patients with high Ki67 expression [29]. Interestingly, *DNHD1* mutations appear to confer a prognostic advantage for lymph-node-positive tumors. Additionally, previous studies have shown that ablation of plectin (PLEC) is associated with impaired cell migration in breast cancer cells, highlighting its potential role in tumor progression [34]. Nonetheless, the clinical significance of other mutations remains unclear, necessitating further functional analysis to characterize their implication in tumor biology and treatment response.

At the transcriptional level, a better response to NAC was associated with the upregulation of genes related to MMR and mTOR signaling pathways and the downregulation of the PI3K-Akt and AGE-RAGE signaling pathways. MMR is well-established for its role in correcting DNA replication errors, which may increase tumor sensitivity to chemotherapy by enhancing genomic stability and reducing the risk of resistance [35]. Similarly, the upregulation of mTOR pathway genes might enhance therapeutic efficacy by promoting cellular adaptation to treatment-induced stress [36]. Conversely, the reduced activity of the PI3K-Akt pathway may lower tumor resistance [37]. The downregulation of the AGE-RAGE pathway, which is linked to inflammation and cellular stress responses, suggests a decrease in inflammatory responses that might otherwise contribute to resistance or tumor survival [38]. Consistent with previous findings that tumor-associated lymphocytes predict response to NAC, our analysis of immune cell composition revealed that baseline pCR tumors had significantly higher fractions of memory B cells and resting NK cells, along with lower proportions of activated mast cells and CAFs. The increased infiltration of memory B cells and resting NK cells in the TME may indicate a readiness for activation, contributing to tumor eradication. In contrast, the lower abundance of activated mast cells and CAFs, which are often associated with tumor progression and immune evasion, may correlate with a favorable response to NAC. Additionally, the limited response to NAC in non-pCR patients could be linked to less effective tumor clearance, possibly attributed to a reduction in cytokine and immune receptor activities. Furthermore, the significant reduction in cytotoxic cell populations in post-NAC TME of pCR patients suggests that an effective response might involve the initial activation and subsequent reduction of these cells as they eliminate tumor cells.

Accumulating evidence suggests that plasma ctDNA is a promising non-invasive prognostic biomarker, with longitudinal undetectable ctDNA indicating a potentially cured population with improved survival outcomes in solid tumors. However, the role of preoperative ctDNA in BC remains unclear, which holds the potential for identifying patients who may not benefit from NAC or who may develop resistance, thereby avoiding unnecessary treatment. Our findings indicate that ctDNA positivity during and after NAC accurately reflects tumor burden and the pathological response assessed in surgical samples, with slightly better performance observed at the P3 time point. Worth noting that two patients (P40 and P44) who were ctDNA-negative at P1 exhibited a positive ctDNA status at P2. This shift could result from chemotherapy-induced cell death or tumor lysis, leading to the release of more ctDNA into the bloodstream. Alternatively, the emergence of resistant tumor clones may also contribute to the increased ctDNA levels. Besides, five patients who were ctDNA-negative at P2 but became ctDNA-positive at P3 were classified as non-pCR during pathological assessment, with concordant results confirmed by clinical response evaluation using radiological findings. In terms of survival, undetectable ctDNA in longitudinal plasma samples appeared to be associated with prolonged DFS and OS. However, landmark survival analyses at the pre-NAC, during-NAC, and post-NAC time points did not show statistically significant differences in survival between ctDNA-positive and ctDNA-negative groups. Several factors may contribute to this observation. First, the relatively small sample size and low event rate may have limited the statistical power to detect survival differences. Second, breast cancer is a biologically heterogeneous disease, and the prognostic value of ctDNA may vary substantially across intrinsic subtypes, which were not evenly represented in our study. Third, variability in adjuvant therapy strategies may have further confounded the association between ctDNA status and clinical outcomes. Lastly, the timing and frequency of blood sampling may have missed dynamic changes in ctDNA that could better capture minimal residual disease or early relapse signals. Overall, while ctDNA dynamics may provide insight into treatment response, their prognostic value in the neoadjuvant setting of breast cancer requires further validation in larger, prospective studies with standardized protocols and extended follow-up.

In our investigation of prognostic biomarkers for long-term survival, we found that patients with baseline *USH2A* mutations have significantly shorter DFS and OS compared to their wild-type counterparts. *USH2A*, a protein-encoding gene primarily implicated in Usher syndrome type IIa and retinitis pigmentosa, was previously associated with poor prognosis in colon adenocarcinoma [39, 40]. Our analysis of gene expression profiles in baseline tumors revealed that *MSL1* was significantly upregulated in *USH2A*-mutated tumors. Notably, upregulation of *MSL1* has been shown to protect cells from DNA damage-induced apoptosis and promote cell survival against DNA-damaging agents [41]. We, therefore, hypothesized that the poor prognosis observed in *USH2A*-mutated patients may be due to elevated *MSL1* expression, which could protect cancer cells from the cytotoxic effects of chemotherapeutic agents. Additionally, *USH2A* mutations have been associated with increased neoantigen loads in lung adenocarcinoma and colon adenocarcinoma [40, 42]. Thus, it is plausible that in BC, patients with these mutations might benefit from immune checkpoint blockade therapies, though further clinical studies are needed before drawing definitive conclusions. Notably, our study is the first to reveal that, despite an initial response to NAC, higher *NHSL1* expression was associated with a worse prognosis in BC, a finding further corroborated by results from the clinical validation cohort. This seemingly paradoxical outcome highlights the complexity of cancer biology. Cell migration, a hallmark of cancer, is crucial for cancer metastasis. Previous research by Law et al. demonstrated that NHSL1 inhibits the Scar/WAVE complex, thereby reducing Arp2/3 activity, which is involved in the stability of lamellipodia and, consequently, cell migration efficiency [43]. We propose that elevated *NHSL1* expression in BC patients may initially reduce cancer cell migration, leading to a better early response to NAC. However, the same mechanism regulating cell migration could facilitate metastasis over time, contributing to a worse long-term survival despite the early success with NAC. Further mechanistic studies are needed to elucidate the functional implications of *NHSL1* in breast cancer.

This study had some limitations. First, the subgroup analysis aimed at identifying predictive and prognostic biomarkers in the neoadjuvant setting of BC patients was constrained by a limited sample size. For example, while on-treatment ctDNA status showed potential as a promising biomarker for predicting survival in patients receiving NAC, the limited sample size posed a significant challenge in drawing definitive conclusions. Second, tumor subtyping was not comprehensively explored due to the retrospective nature of the study and the small number of cases in certain subgroups. Consequently, validation in larger, prospective cohorts with adequate representation of intrinsic tumor subtypes is warranted. Third, the analysis of ctDNA dynamics was limited to preoperative assessment for potential associations with NAC responses. Including postoperative longitudinal time points during follow-up could offer a more comprehensive view of ctDNA-based minimal residue disease and its prognostic implications. Lastly, future studies leveraging external NAC cohorts with both genomic and transcriptomic data will be instrumental in further validating and extending the current findings.

In conclusion, our comprehensive genomic and transcriptomic analysis identified promising biomarkers that may predict tumor responses and survival outcomes in BC patients who received neoadjuvant chemotherapy followed by surgery. This study highlights the clinical significance of early assessment of treatment response for developing individualized treatment strategies, which may ultimately translate into long-term survival benefits for patients.

## Supplementary Information

Supplementary Material 1.

Supplementary Material 2.

## Data Availability

The datasets generated and/or analyzed during this current study are available from the corresponding author upon reasonable request.
