# Supplementary material for: Unraveling breast cancer response to neoadjuvant chemotherapy through integrated genomic, transcriptomic, and circulating tumor DNA analysis
Source: Breast Cancer Res. 2025 May 1;27:64. doi: 10.1186/s13058-025-02026-5 (PMC12044986; doi:10.1186/s13058-025-02026-5)
Supplement: Supplementary file 2 — Supplementary Material 2. [file 13058_2025_2026_MOESM2_ESM.docx]

**Supplementary Materials**

## **Supplementary Tables**

| **Table S1. Univariate analysis for baseline clinicopathological features and survival outcomes** | | | | |
| --- | --- | --- | --- | --- |
|  | Disease-free survival | | Overall survival | |
| Variable | HR (95% CI) | *P*-value | HR (95% CI) | *P*-value |
| Clinical stage (III vs. II) | 6.09 (1.45-25.56) | 0.014* | 6.71 (0.61-74.14) | 0.120 |
| *CHD8* mutation | 4.43 (1.05-18.68) | 0.043* | 10.27 (0.93-113.44) | 0.057 |
| *HMCN1* mutation | 5.52 (1.37-22.2) | 0.016* | 1.97 (0.18-21.78) | 0.579 |
| *SULF1* mutation | 4.43 (1.05-18.68) | 0.043* | 10.27 (0.93-113.44) | 0.057 |
| *UNC80* mutation | 4.43 (1.05-18.68) | 0.043* | 10.27 (0.93-113.44) | 0.057 |
| *USH2A* mutation | 11.85 (2.77-50.81) | 0.001*** | 8.42 (0.76-92.94) | 0.082 |
| Abbreviations: HR, hazard ratio; CI, confidence interval | | | | |

| **Table S2. AUC of baseline gene expression for predicting pathological response** | | |
| --- | --- | --- |
| Baseline expression | AUC (95% CI) | Description |
| *GYLTL1B* | 0.93 (0.84-1) | Involved in protein O-linked mannosylation |
| *SOX6* | 0.90 (0.78-1) | Transcriptional activator required for CNS development, chondrogenesis and maintenance of cardiac and skeletal muscle cells |
| *CYB5R2* | 0.89 (0.78-1) | Involved in cholesterol biosynthesis, fatty acid desaturation and elongation, and respiratory burst in neutrophils and macrophages |
| *TRDMT1* | 0.89 (0.69-1) | Methylation of aspartic acid transfer RNA |
| *DLGAP1* | 0.88 (0.76-1) | Predicted to enable molecular adaptor activity |
| *PM20D2* | 0.88 (0.75-1) | Enables dipeptidase activity and identical protein binding activity |
| *MID1* | 0.87 (0.73-1) | Formation of homodimers acting as anchor points to microtubules |
| *DDX26B* | 0.85 (0.66-1) | Predicted to be involved in snRNA 3'-end processing |
| *PSAT1* | 0.84 (0.7-0.99) | Encodes a phosphoserine aminotransferase |
| *L3MBTL4* | 0.84 (0.67-1) | Predicted to enable chromatin binding activity and histone binding activity |
| *TAF4B* | 0.82 (0.63-1) | Encodes a TBP-associated factor that may be responsible for mediating transcription by a subset of activators in B cells |
| *PRKX* | 0.81 (0.61-1) | Involved in macrophage and granulocyte maturation |
| *KIAA0020* | 0.80 (0.62-0.98) | Enables RNA binding activity |
| *CENPW* | 0.80 (0.61-0.99) | Predicted to enable DNA binding activity and protein heterodimerization activity. Involved in chromosome segregation, kinetochore assembly, and mitotic cell cycle. |
| *RCL1* | 0.78 (0.56-1) | Enable endoribonuclease activity |
| *PSRC1* | 0.78 (0.53-1) | Encodes a target for regulation by TP53 |
| *ZNF695* | 0.76 (0.55-0.98) | Predicted to enable DNA-binding transcription factor activity, RNA polymerase II-specific and RNA polymerase II cis-regulatory region sequence-specific DNA binding activity |
| *LBR* | 0.76 (0.51-1) | Encodes a protein that mediates interaction between chromatin and lamin B |
| Abbreviations: AUC, area under the curve; CI, confidence interval | | |

| **Table S3. Univariate analysis of baseline gene expression for survival outcomes** | | | | |
| --- | --- | --- | --- | --- |
|  | Disease-free survival | | Overall survival | |
| Gene (High vs. Low) | HR (95% CI) | *P*-value | HR (95% CI) | *P*-value |
| *NHSL1* | 4.7 (1.05-21.13) | 0.043* | 8.76 (0.91-84.24) | 0.060 |
| *TRMT11* | 4.4 (0.98-19.7) | 0.053 | 2.79 (0.39-19.8) | 0.305 |
| *REPS1* | 4.4 (0.98-19.7) | 0.053 | 2.79 (0.39-19.8) | 0.305 |
| *CDCA3* | 4.29 (0.96-19.22) | 0.057 | 3.05 (0.43-21.68) | 0.266 |
| *ZNF695* | 4.29 (0.96-19.22) | 0.057 | 3.05 (0.43-21.68) | 0.266 |
| *COLEC12* | 4.05 (0.91-18.11) | 0.067 | 3.05 (0.43-21.68) | 0.266 |
| *LBR* | 2.54 (0.57-11.38) | 0.223 | 9.16 (0.95-88.21) | 0.055 |
| *RAVER2* | 2.54 (0.57-11.38) | 0.223 | 9.16 (0.95-88.21) | 0.055 |
| Abbreviations: HR, hazard ratio; CI, confidence interval | | | | |

## **Supplementary Figures**


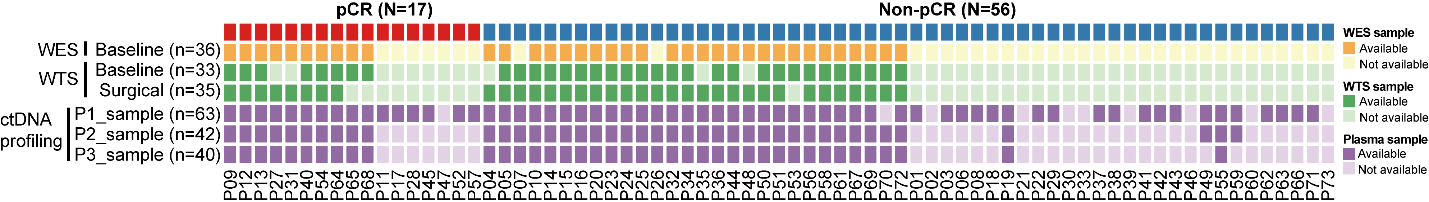


### **Figure S1. Sample availability for the study cohort**

The heatmap displays the availability of data across various analyses for each of the 73 patients. Each column represents a patient, and each row corresponds to a specific sample. ctDNA profiling was performed on plasma samples collected at three time points: before NAC (P1), during NAC (P2), and after NAC (P3).


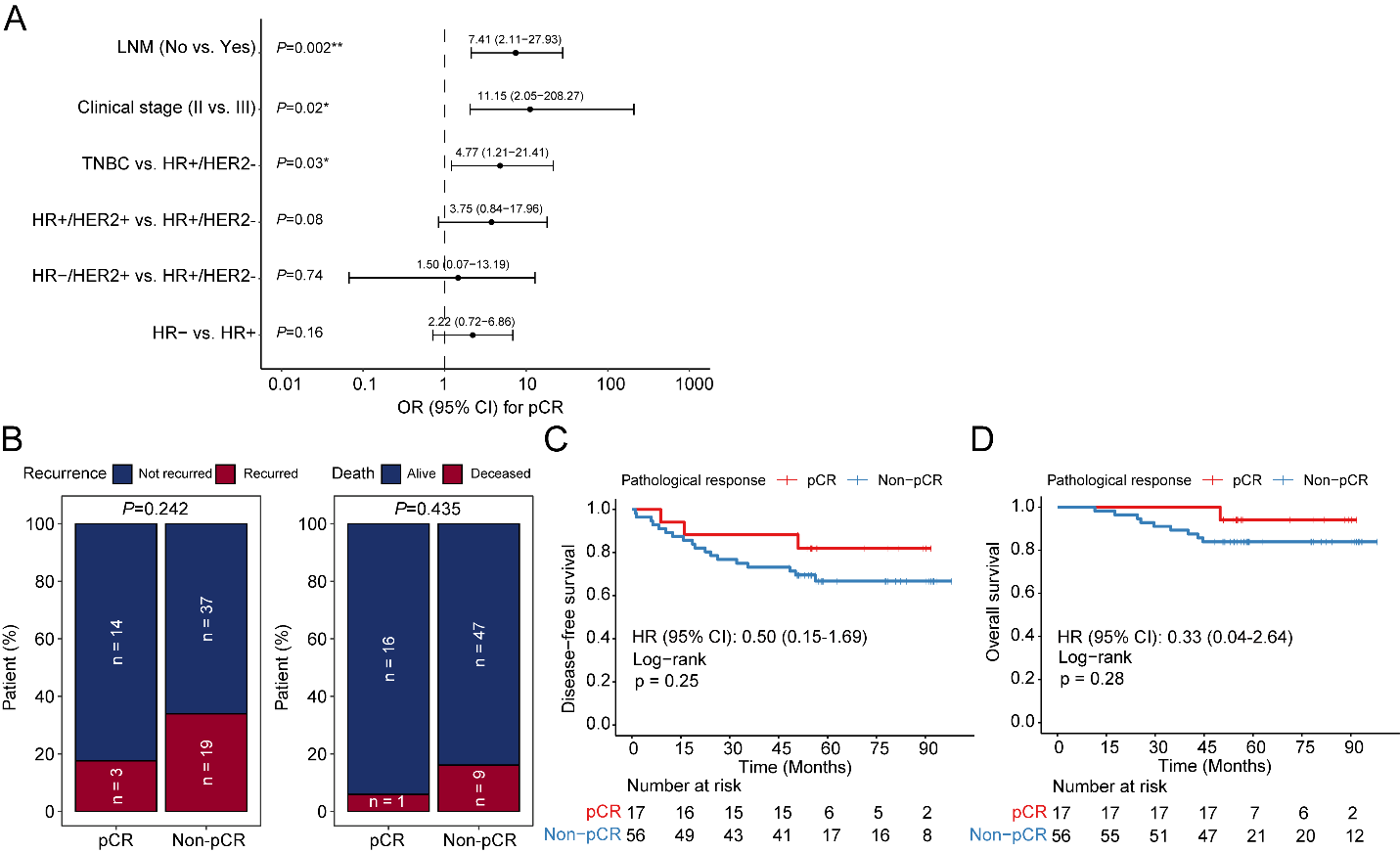


### **Figure S2. Pathological complete response predicts long-term outcomes**

(**A**) Odds ratio (OR) and 95% confidence intervals (CI) for the association between clinical features and pathological complete response (pCR) following neoadjuvant chemotherapy. (**B**) Bar plots showing the proportion of patients in subgroups, stratified by pathological response, who experienced disease recurrence or death. (**C, D**) Kaplan-Meier curves illustrate the disease-free survival (C) and overall survival (D) in pCR and non-pCR patients within the final evaluable population.


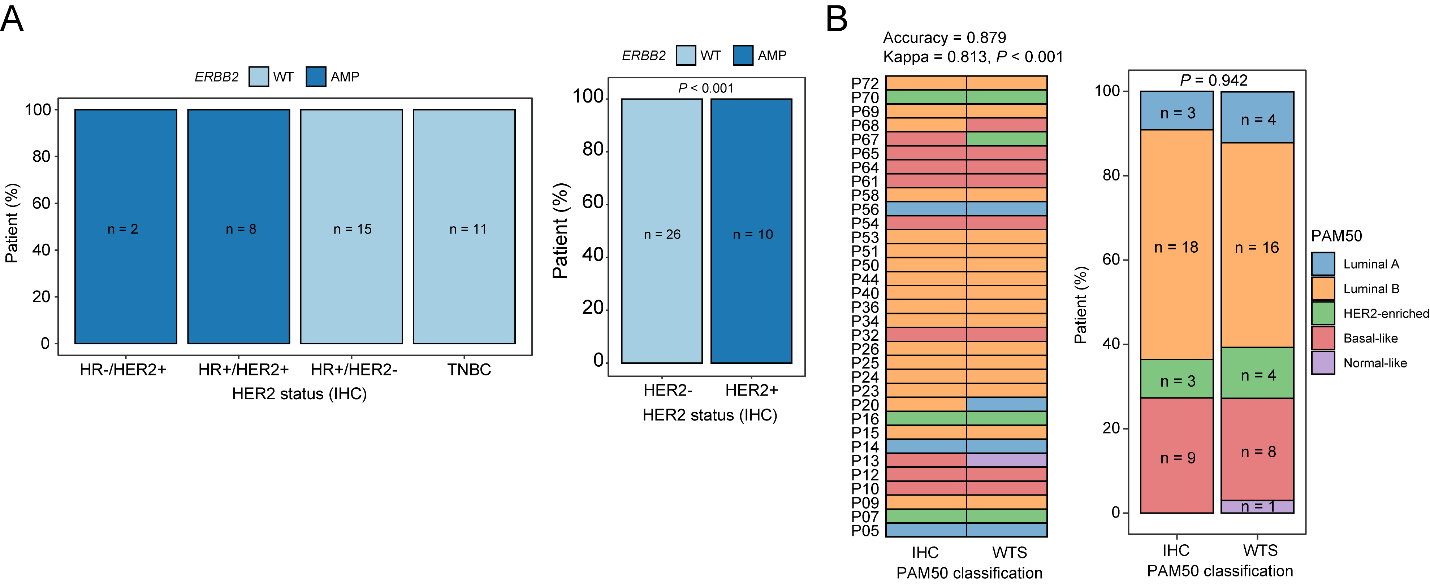


### **Figure S3. Concordance analysis of NGS- and IHC-based methods for cancer subtyping**

(**A**) *ERBB2* copy number variation status across immunohistochemistry (IHC)-defined receptor subtypes in baseline tumor samples. (**B**) Concordance analysis of subtype classification between IHC and whole transcriptome sequencing (WTS)-based PAM50 profiling. The heatmap displays subtype classifications for each patient using both methods, with accuracy and kappa coefficient depicted. Stacked bar plots illustrate the distribution of PAM50 subtypes according to each classification approach.


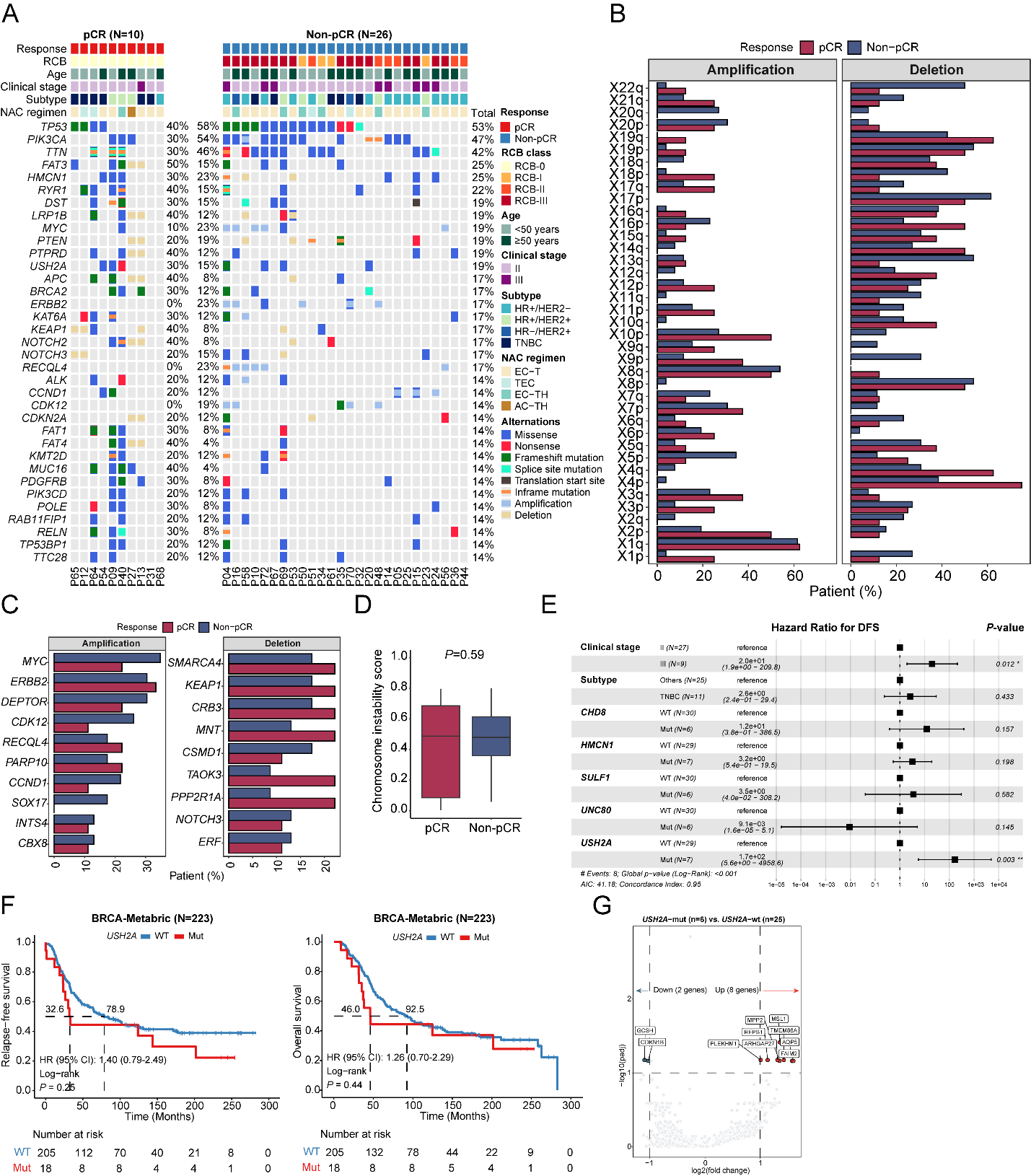


### **Figure S4. Genetic landscape of patients before NAC**

(**A**) Co-mutation plot of genetic variants in baseline tumors (N=36). Patients were grouped by pathological response and each column represents one patient. (**B, C**) Bar plot showing the proportion of patients harboring arm-level (B) and focal-level (C) copy number variants in baseline tumors. (**D**) Box plot illustrating the chromosome instability score in pathological complete response (pCR) and non-pCR patients. (**E**) Forest plot depicting the multivariate analysis of the association between clinicopathological features and disease-free survival (DFS) in baseline tumors. (**F**) Kaplan-Meier curves illustrate the disease-free survival and overall survival of patients with or without baseline *USH2A* mutations in the BRCA-Metabric cohort (N=223). (**G**) Volcano plot depicting the differentially expressed genes (DEGs) between *USH2A*-mutated and *USH2A*-wildtype baseline tumors. Due to minimal changes in expression between subgroups, DEGs were defined using a threshold of log2 (fold change) > ±1 and an FDR-adjusted p-value (padj) < 0.1.


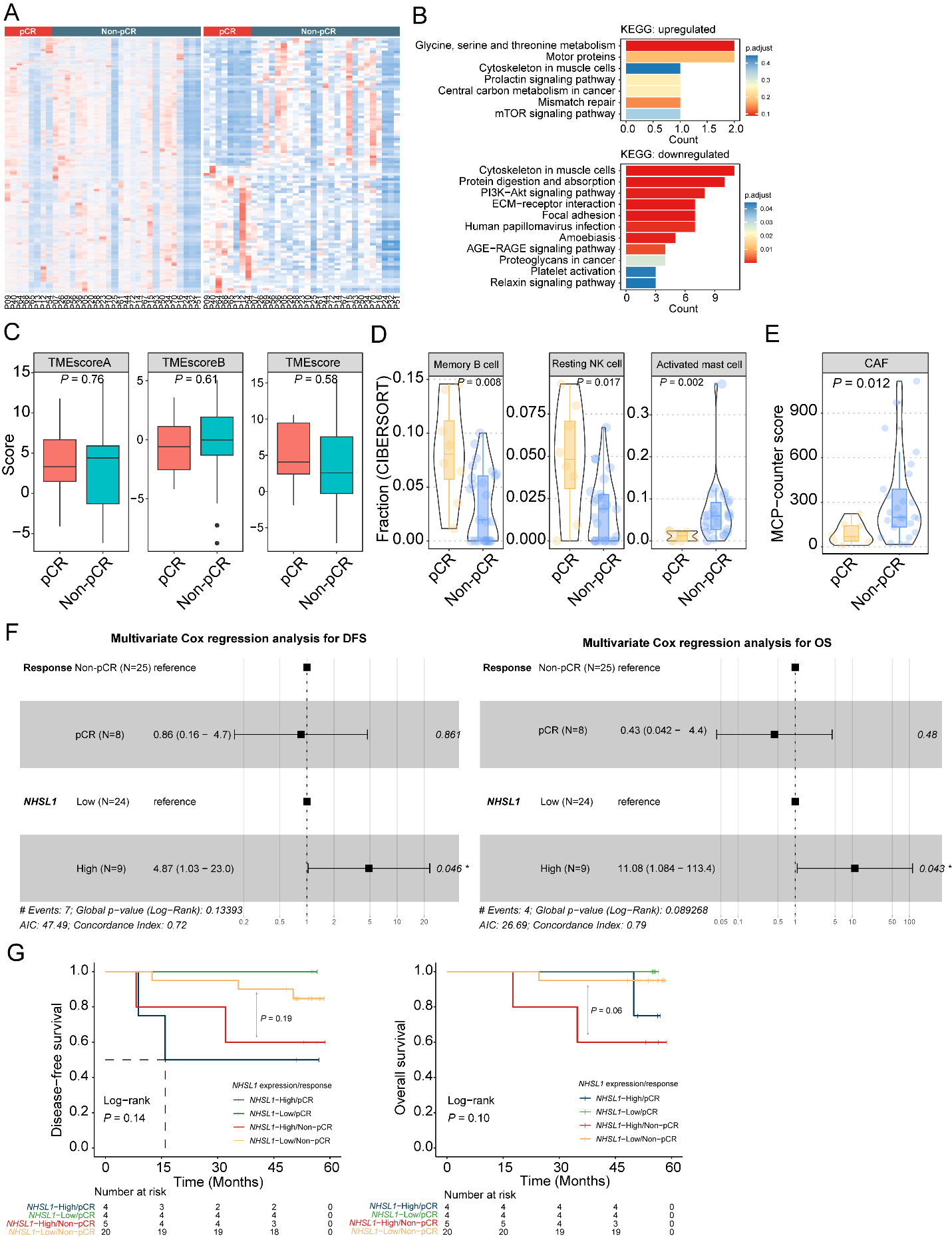


### **Figure S5. Gene expression profiling of patients before NAC**

(**A**) Heatmap of all genes (top) and differentially expressed genes (DEGs) in baseline tumor samples. (**B**) Functional enrichment of DEGs through KEGG analysis. (**C**) Box plots illustrating TME scoring in pathological complete response (pCR) and non-pCR tumors. (**D**) Relative fraction of selective immune cell populations in baseline tumors grouped by pathological response. (**E**) MCP-counter scores of cancer-associated fibroblasts (CAF) in baseline tumors. (**F**) Forest plots showing the multivariate Cox regression analysis for disease-free survival and overall survival incorporating pathological response and *NHSL1* expression status. (**G**) Kaplan-Meier curves show the disease-free survival and overall survival of patients stratified by both baseline *NHSL1* expression and pathological response status.


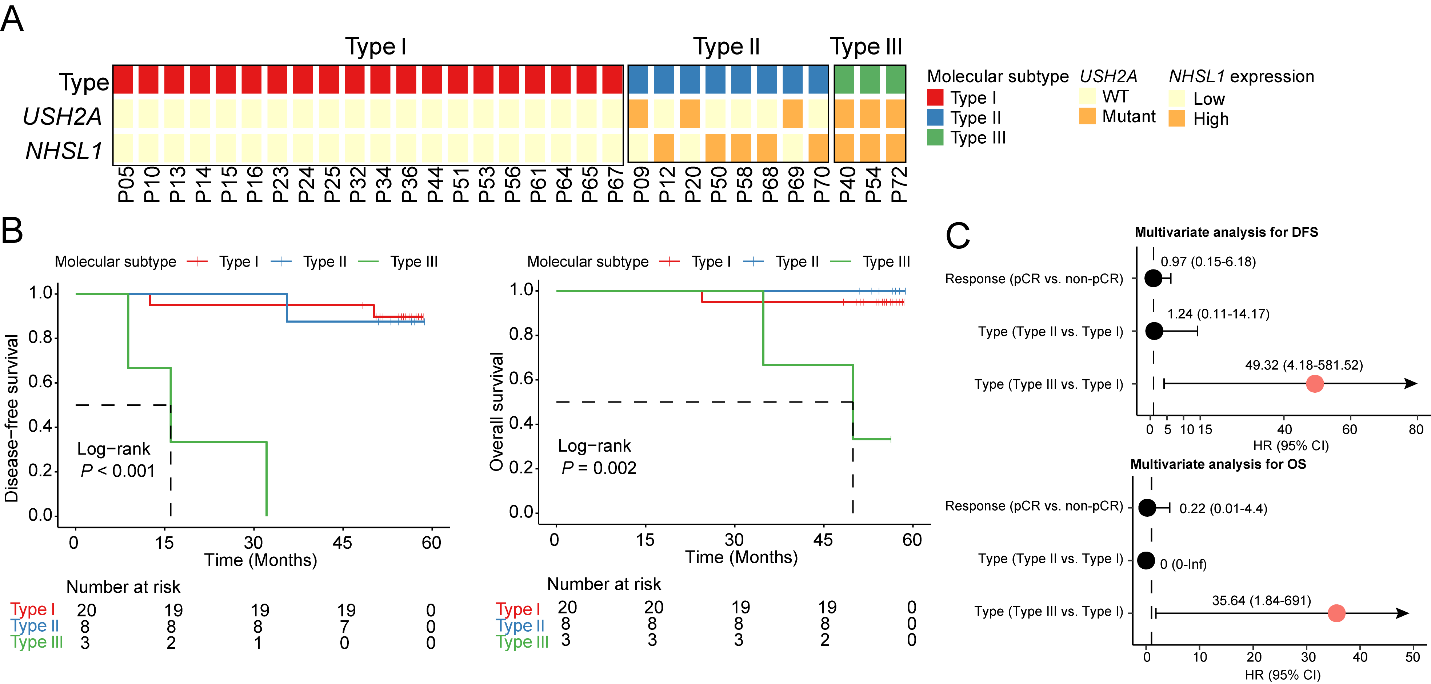


### **Figure S6. Molecular subtyping identified patients at high risk of recurrence and progression**

(**A**) Molecular subtyping of breast cancer patients receiving neoadjuvant chemotherapy by baseline *USH2A* mutations and *NHSL1* expression. (**B**) Kaplan-Meier curves showing the disease-free survival (DFS) and overall survival (OS) of patients stratified by molecular subtypes. (**C**) Multivariate Cox regression analysis for DFS and OS, incorporating pathological response and molecular subtype.


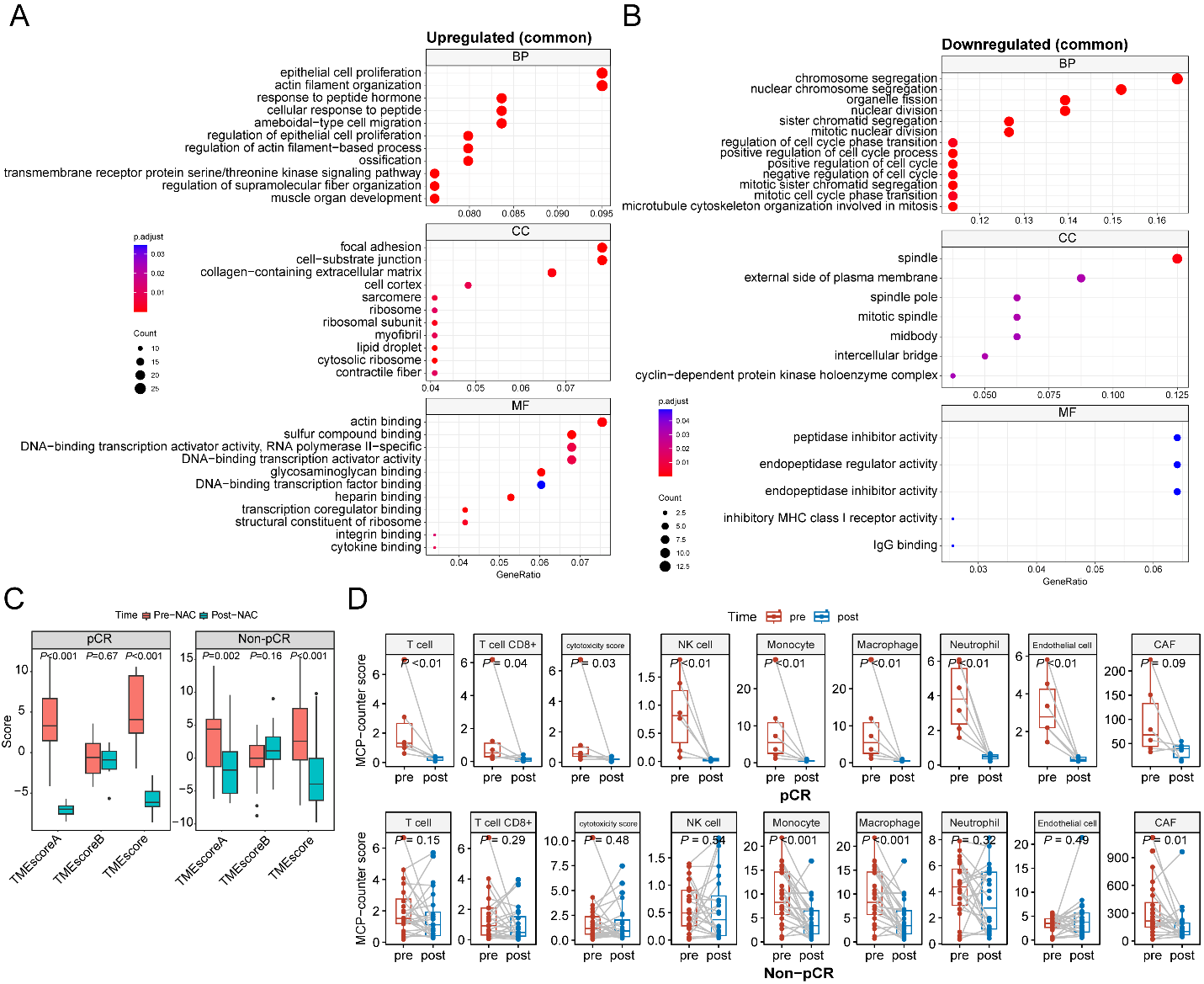


### **Figure S7. Chemotherapy-related changes at the transcriptional level**

(**A, B**) Gene Ontology analysis of genes with altered expression in both pathological groups. (**C**) Box plots comparing the TME scores in surgical and baseline tumors of patients categorized by pathological response. (**D**) MCP-counter scores of selective immune cell populations in tumor samples before and after neoadjuvant chemotherapy.


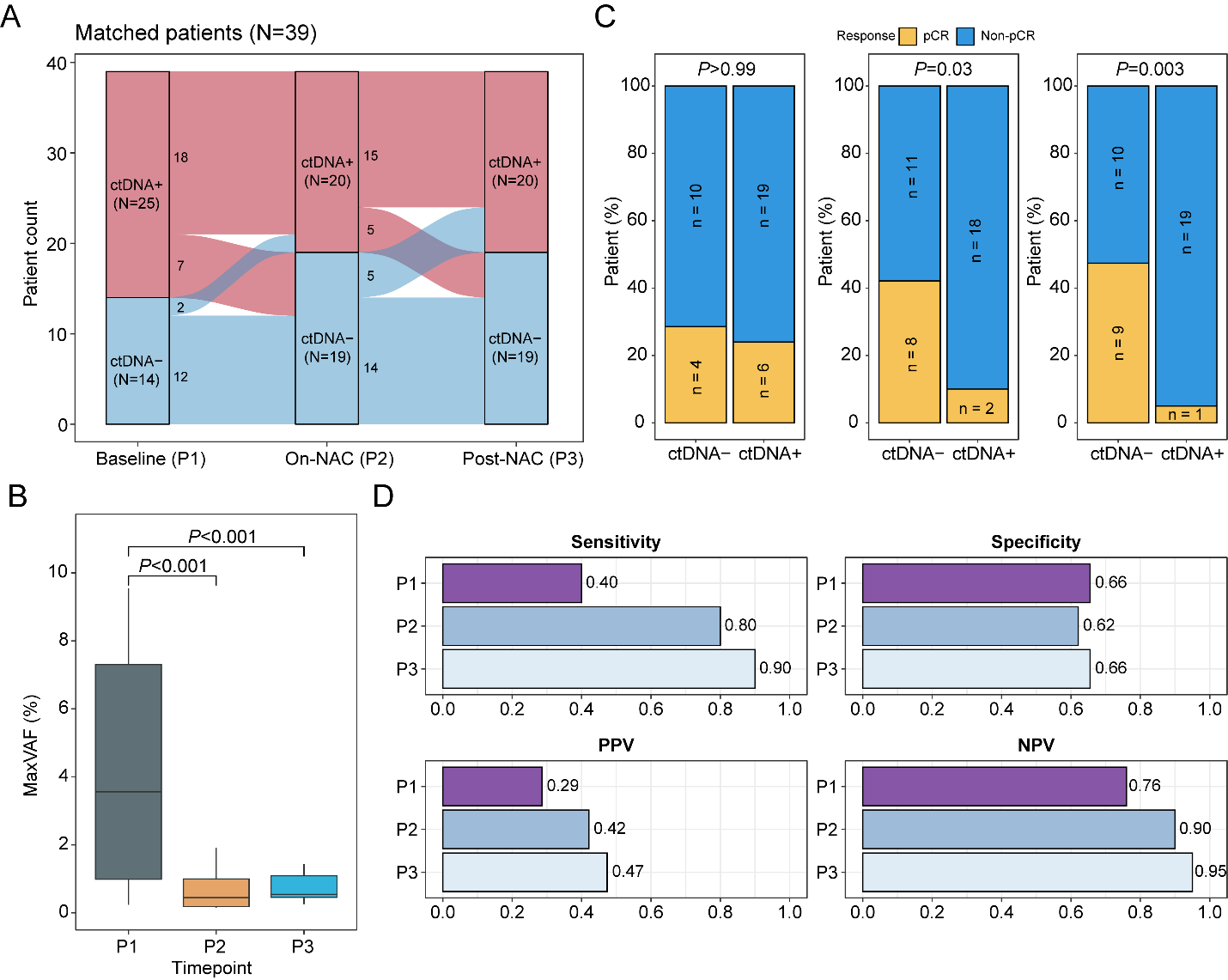


### **Figure S8. Dynamic ctDNA changes in patients with matched samples**

(**A**) Sankey plot depicting changes in the proportion of patients stratified by ctDNA status at baseline, on-NAC, and post-NAC time points. (**B**) Box plot showing the change in the maximum variant allele frequency (maxVAF) in plasma ctDNA during NAC. (**C**) Bar plots showing the proportion of patients achieving pathological complete response (pCR) or non-pCR, stratified by ctDNA status at the three time points. (**D**) Performance analysis evaluating the predictive value of undetectable ctDNA at each of the three time points for predicting tumor response to NAC. NAC, neoadjuvant chemotherapy; PPV, positive predictive value; NPV, negative predictive value.


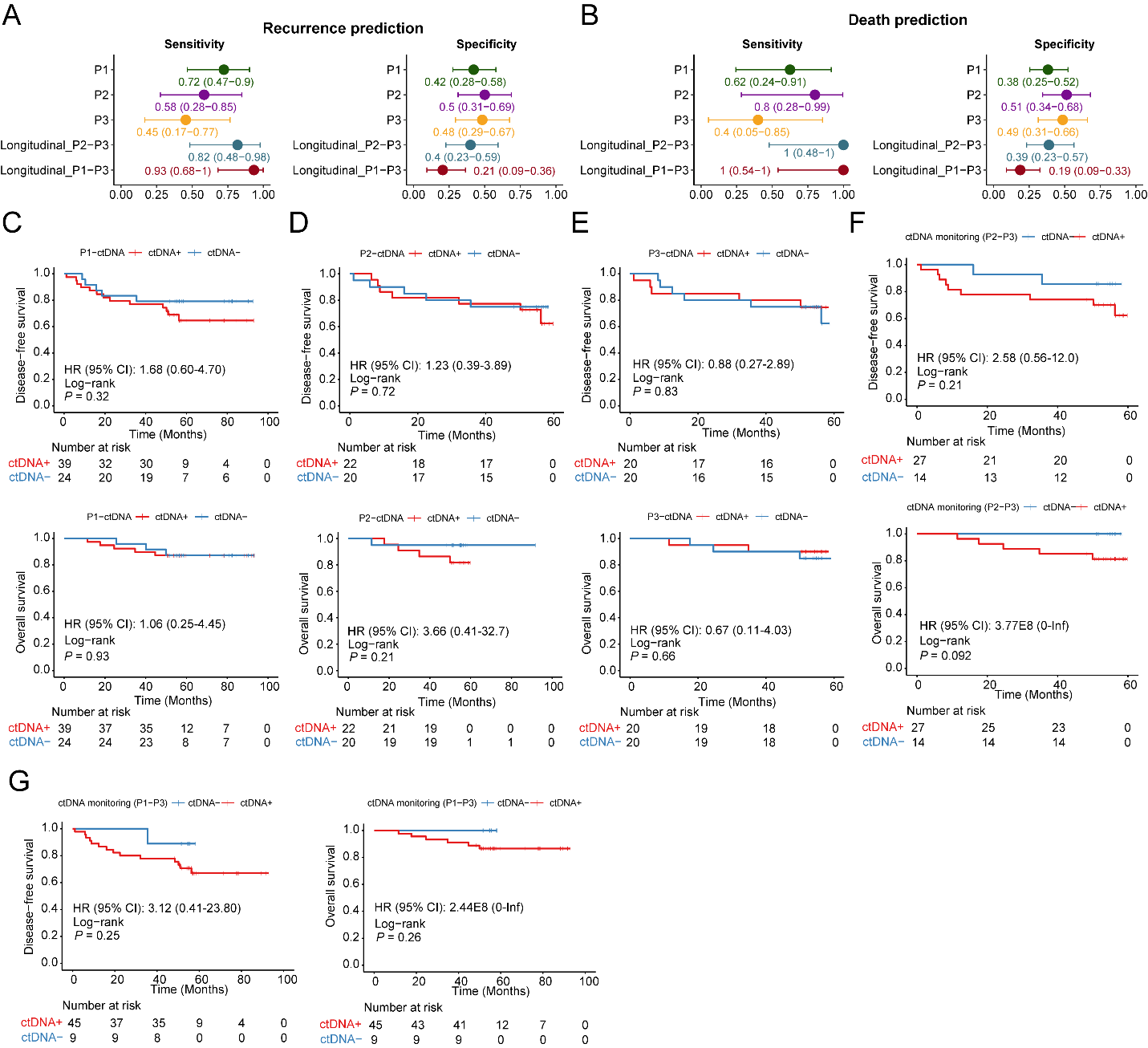


### **Figure S9. ctDNA positivity status and patient prognosis**

(**A, B**) Predictive value of landmark ctDNA and longitudinal ctDNA status for disease recurrence (A) and death (B). (**C-E**) Kaplan-Meier curves showing the disease-free survival and overall survival stratified by ctDNA status at pre-NAC (P1), during NAC (P2), and post-NAC (P3) time points. (**F, G**) Kaplan-Meier curves showing the disease-free survival and overall survival, stratified by longitudinal ctDNA status from P2 to P3 time points (F) and P1 to P3 time points (G).
